# Insights into Adsorption Characterization of Sulfated Xylans onto Poly(ethylene terephthalate)

**DOI:** 10.3390/polym12040825

**Published:** 2020-04-05

**Authors:** Lidija Fras Zemljič, Nena Dimitrušev, Rok Zaplotnik, Simona Strnad

**Affiliations:** 1 Institute of Engineering Materials and Design, Faculty of Mechanical Engineering, University of Maribor, Smetanova ulica 17, 2000 Maribor, Slovenia; lidija.fras@um.si; 2 Centre of Sensor Technology, Faculty of Mechanical Engineering, University of Maribor, Smetanova ulica 17, 2000 Maribor, Slovenia; nena.dimitrusev@um.si; 3 Plasma Laboratory, Department of Surface Engineering and Optoelectronics, Jozef Stefan Institute, Jamova 39, SI-1000 Ljubljana, Slovenia; rok.zaplotnik@ijs.si

**Keywords:** adsorption, sulfated xylan, poly(ethylene terephthalate), quartz crystal microbalance (QCM-D), antithrombotic polymer composite material

## Abstract

The main aim of this investigation was to study the interaction of sulfated xylans as antithrombotic substances with poly(ethylene terephthalate) (PET) model films as a model for blood contacting surfaces. The adsorption of sulfated xylans onto PET model films was studied as a function of pH and ionic strength using the quartz crystal microbalance with dissipation (QCM-D) technique. The application of positively charged polyethyleneimine (PEI) as an anchoring polymer was done to improve the adsorption. The hydrophilic/hydrophobic properties of functionalized PET surfaces were monitored by goniometry, whilst their elemental composition was determined by X-ray photoelectron spectroscopy. Sulfated xylans adsorbed favorably at pH 5 by physical interactions and by entropy gain driven adsorption. Higher ionic strengths of solutions improved adsorption, due to the reduction of electrostatic repulsive forces between PET surfaces and anionic xylans’ macromolecules. The intermediate PEI layer caused more extensive and stable adsorption due to Coulomb interactions. The surface modifications presented in this work provided important information regarding the adsorption/desorption phenomena between antithrombotic sulfated xylans and PET surfaces. The latter is of great interest when preparing advanced polymer composite material such as functional antithrombotic PET surfaces for blood-contacting medical devices and presents an extremely challenging research field.

## 1. Introduction

The development of advanced polymer hemocompatible biomaterials is of extreme importance, due to the fact that cardiovascular diseases and their complications are among the main reasons for deaths in the developed world [1,2]. The main complications that occur in vascular grafts are reduced patency due to intimal hyperplasia or thrombus formation. Thrombotic occlusion after vascular reconstructive surgery is still a frequent complication, especially when small diameter vascular prostheses are involved. To overcome these problems, currently, most vascular grafts inner surfaces are coated by heparin, the widely applied antithrombotic substance [3]. However, it is well known that heparin could cause some side effects, such as thrombocytopenia and abnormal bleeding in treated patients [4,5]. Moreover, as a substance derived from mammalian sources, heparin might be contaminated with animal proteins or pathogenic agents that could cause allergies or some immunity disorders [6]. 

To avoid the risk of contamination with pathogenic agents, nowadays, the research is focused on therapeutics derived from non-mammalian, mostly plant, sources [7]. Among them, polysaccharides are extremely remarkable, because of their structural specifics and wide functionalization prospects [8,9,10]. Sulfated polysaccharides, that are either of natural, semisynthetic or synthetic origin, have shown multiple biological activities; especially, their anticoagulant activity has been studied widely [9,11,12,13]. Several methods have been reported for polysaccharide sulfation in order to introduce anticoagulant properties [14,15]. 

Xylans are heteropolymers with a β-(1-4)-D-xylopyranose backbone, which is branched by short carbohydrate chains comprising: D-glucuronic acid or its 4-O-methyl ether, L-arabinose and/or various oligosaccharides [16]. Such polysaccharide structures provide an interesting platform for different functionalization and derivatization in order to achieve water solubility, antioxidant activities or antiviral properties [17,18,19]. Moreover, xylans are the main hemicellulose component of secondary cell-walls, constituting about 20%–30% of the biomass of dicotyl plants and, in some tissues of monocotyl plants; they occur up to 50% [20]. Hemicelluloses are found as organic wastes or by-products of renewable forest and agricultural products, however, unlike cellulose, they have not yet found broad industrial application [20,21]. The development of new, high-added value products made of hemicelluloses are, therefore, extremely stimulating. 

In recent decades, great interest has been pointed toward xylans as renewable polymers in food [22,23], medical [24,25] and pharmaceutical applications [26,27]. Recently, a wide study of sulfation procedures for xylans from different sources was performed, and broad structural varieties of xylan sulfates were obtained and their structure analyzed [15,28,29]. It has also been shown that sulfated xylans are prospective antithrombotic agents [30,31]. However, for their application as a coating substance for medical devices, such as vascular grafts, vascular stents, heart valve cuffs, etc. which are mostly made from poly(ethylene terephthalate) (PET), it is of great significance to understand clearly the interactions between a substrate material and an adsorbate; i.e., the adsorption/desorption phenomena. Optimizing interactions between PET and anionic xylans is of paramount importance for ensuring the resistance and efficiency of functional coatings on materials’ surfaces in the harsh conditions of blood contacting biological environments. 

In our previous work [32], where the study regarding the interaction of carboxymethylated xylans with poly(ethylene terephthalate) (PET) surfaces using QCM-D was presented, it has been pointed out that optimal conditions for stable and efficient carboxymethylated xylan adsorption for PET materials with final hydrophilic character may be precisely established. With this knowledge behind, it became clear that with similar approach of interaction study between sulfated xylans and poly(ethylene therephtalate) (PET), the optimal chemical conditions for adsorption and for the accurate design of functional coatings for blood-contacting medical devices, might also be developed. Hitherto, to the best of the authors’ knowledge no such complex and detailed study of using sulfated xylans as polysaccharide coating for PET functionalization has been presented previously. 

Thus, in the present paper, the interactions between model PET surfaces and sulfated glucorono- and arabino- xylans were studied by means of quartz crystal microbalance with dissipation (QCM-D). The adsorption processes were accurately examined, such as the dependence on pH, ionic strength as well as the use of the anchoring polyethyleneimine (PEI) layer. The results obtained in this study are important for expanding the potential use of these anionic polysaccharides integrated into advanced composite materials designed to improve or enhance the functionality of medical devices and, consequently, to extend their use. 

## 2. Materials and Methods 

### 2.1. Materials

#### 2.1.1. Xylans

4-O-methyl glucuronoxylan from beech wood (BXL with *M*_w_ = 22,300 g/mol, *Mw*/*Mn* = 1.4) was obtained by the extraction of beech wood holocellulose. The holocellulose was prepared by treatment of the starting material with 4.5% sodium chlorite for 120 h at room temperature [33]. 

Arabinoxylan from oat-spelts (OX) (*Mw* = 23,500 g/mol; *Mw*/*Mn* = 2.3) was obtained by sodium hydroxide extraction and precipitation, as published previously [34]. 

#### 2.1.2. Poly(ethylene terephthalate)

For the preparation of PET model films poly(ethylene terephthalate) (PET) foil Mylar^®^ (Dupont, DuPont Teijin Films UK Ltd., Redcar, UK) was applied with a thickness of 175 μm. Prior to application it was immersed into ethanol (*M_W_* = 46.07 g/mol, 99.8% (GC) from Sigma-Aldrich, St. Louis, MO, USA) and cleaned in an ultrasonic bath for 10 min, washed thoroughly with demineralized water, and air-dried.

### 2.2. Sulfation of Xylan Samples

Xylan samples were sulfated according to the procedure of Martinichen-Herrero et al. [14]. The samples were labeled as follows: BXLS for sulfated glucuronoxylan and OXS for sulfated arabinoxylan. The chemical structure of sulfated xylans was represented in our former publication [35].

### 2.3. FTIR Spectroscopy

FT-IR analysis was performed using a Perkin Elmer spectrum GX FT-IR spectrometer (Bruker, Billerica, MA, USA) with a Golden Gate ATR attachment and diamond crystal. The absorbance measurements were carried out in the range of 400–4000 cm^−1^, repeating 16 scans by the resolution of 4 cm^−1^.

### 2.4. Elemental Analysis

Elemental analysis was made by the Vario EL Cube (Elementar Analysensysteme GmbH, Hanau, Germany) analyzer, at the following processing conditions: temperature of 1150 °C in the combustion tube and 850 °C in the reduction tube, with a helium flow of 230 mL min^−1^ as well as an oxygen flow of 35 mL min^−1^. The detection limit for the analysis of the 5 mg sample was about 0.1 wt % for C, 0.3 wt % for H, 0.02 wt % for N and 0.4 wt % for S. The drying of samples was performed for the time of one week by P_2_O_5_ in a desiccator before elemental analyzing. 

### 2.5. Polyelectrolyte Titrations 

Polyelectrolyte titrations of an aqueous media (xylan solutions) were made at different pH values (pH = 2, 4, 8), previously adjusted with adding of NaOH (0.1 M, Sigma-Aldrich, St. Louis, MO, USA) or HCl (0.1 M, Sigma-Aldrich, St. Louis, MO, USA). For a measurement at a selected pH, 2 mL of dissolved xylan (0.1% in water) was pipetted into a titration vessel and 1 mL of 0.1 mM o-toluidine blue indicator was inserted as well. The sample was diluted by a distillate water, to a final volume of 40 mL and further adjusting of pH. Titrations were performed using cationic polyelectrolyte pDADMAC as a titrant (average *Mw* < 100,000, c ≈ 1 Mm, Sigma-Aldrich, St. Louis, MO, USA), in increments of 100 μL every 3–10 s at a Mettler Toledo DL 53 titrator with a 10 mL burette and room temperature. The absorbance was measured as a potential change in mV, using a Mettler Toledo Phototrode DP660 at a wavelength of 660 nm. The concentration of deprotonated anionic groups (sulfate and carboxyl groups) was determined from the equivalent volume of pDADMAC solution added, detected as the steepest rise in the curve of absorbance vs. volume of pDADMAC, and by assuming a 100% binding stoichiometry between the ammonium and sulfate and carboxyl groups.

### 2.6. Determination of Activated Partial Thromboplastine Time (APTT)

The APTT determination was done by mixing of 80 μL of control plasma N (ORKE 41, Dade Behring) and 20 μL of polysaccharides samples (0–5 μg) dissolved in 0.9% sodium chloride (Sigma-Aldrich, St. Louis, MO, USA) with 100 μL APTT reagent Pathromtin SL (OQGS29, Dade Behring). After incubation of 3 min at 37 °C, 100 μL of pre-warmed 0.025 M CaCl_2_ (ORHO37, Dade Behring) solutions were added to the mixtures in order to initiate a coagulation cascade. Coagulation time was determined by coagulometer Thrombotrack™ Solo (Axis-Shield PoC AS, Oslo, Norway).

### 2.7. Model PET Films Preparation by the Spin-Coating Technique

Spin coated model PET films were prepared by dissolving 1 wt % of PET foil in 1,1,2,2-tetrachloroethane, and heating (*T* ≈ 150 °C) until the foil was dissolved. When the solution was cold, it was filtered through a 0.2 μm Acrodisc GHP filter (Sigma-Aldrich, St. Louis, MO, USA). 30 μL of solution was spread on a 14 mm gold quartz crystal and spin coated at a maximum of 2000 rpm for 60 s.

### 2.8. Adsorption Studies by Quartz Crystal Microbalance with Dissipation (QCM-D)

Adsorption of xylans onto PET films was studied using a quartz crystal microbalance with a dissipation unit (QCM-D) E4 instrument from Q-Sense AB, Gothenburg, Sweden. 

The analysis is based on the measurement of changes in resonance frequency of a thin AT-cut piezoelectric quartz crystal disc [36,37,38]. It allows, simultaneously, measurement of change in resonance frequency and energy losses (dissipation) when the mass adsorbed on an oscillated piezoelectric crystal changes. The resonant frequency (*f_0_* ≈ 5 MHz) of the crystal decreases when additional mass is adsorbed on its surface following the Sauerbrey relationship:

(1) $$\Delta m=-\frac{C\cdot \Delta f}{n}$$

where ∆*m* is the change in mass of the crystal, *C* is the mass sensitivity constant (17.7 ng Hz^−1^ cm^2^ for a 5 MHz quartz crystal), *n* is the overtone number (1, 3, 5, 7, 9, 11, 13), and ∆*f* is the frequency change.

In cases when masses of a soft (i.e., viscoelastic) film are measured, the measure of damping of the crystal oscillation (dissipation factor *D*) is of great importance. The dissipation is defined as:

(2) $$D=\frac{E_{diss}}{2\pi E_{stor}}$$

where *E_diss_* is the energy dissipated and *E_stor_* is the total energy stored in the oscillator during one oscillation cycle.

In order to improve the adsorption and persistence of adsorbed sulfated xylan layers, after rinsing, polyethyleneimine (PEI) was applied as an anchoring agent in a concentration of 0.05%. In order to improve the PEI adsorbed layer stability, after the adsorption of PEI onto the PET surface, the model film’s samples were dried in a vacuum dryer. In the next stage, the adsorption studies of sulfated xylans onto PEI layers were performed, also using QCM.

### 2.9. X-ray Photoelectron Spectroscopy (XPS) 

XPS spectra of prepared samples were recorded using the PHI model TFA XPS spectrometer at the Josef Stefan Institute, Ljubljana, Slovenia. XPS spectra were recorded by a PHI TFA XPS from Physical Electronics, Chanhassen, MN, USA, in order to evaluate the surface of the samples. The base pressure in the XPS analysis chamber was around 6 × 10^−8^ Pa. The samples were agitated with X-rays terminated at 400 µm spot area with monochromatic Al *K*_α1,2_ radiation (1486.6 eV) at 200 W. Photoelectrons were detected with a hemispherical analyzer, at an angle of 45° position with respect to the sample surface. The energy resolution was about 0.6 eV. Spectra were recorded from at least three locations for each sample and surface elemental concentrations were calculated from the survey-scan spectra using the Multipak software.

### 2.10. Wettability and Contact Angle Measurements

The Dataphisics OCA 35 (DataPhysics Instruments GmbH, Filderstadt, Germany) Contact Angle measurement system was used for the determination of the Surface Contact Angles (SCA). The sessile drop method was performed at room temperature with a drop of MQ water volume of 3 μL, and at least three times on each surface. The average values were calculated. 

## 3. Results

### 3.1. Efficiency of the Sulfation Procedure

In order to confirm the efficiency of sulfation procedures, FTIR spectroscopy and elemental analyses were performed for non-modified and sulfated glucuronoxylan and arabinoxylan-samples. Due to the fact that sulfate groups are one of the main sources of polymer antithrombotic properties [14], it is of high importance to be able to determine the amount of those groups precisely, especially when sulfated polysaccharides act as an adsorbate for solid matrices [9,38]. Polyelectrolyte titrations were used for this purpose. 

**Figure 1:**
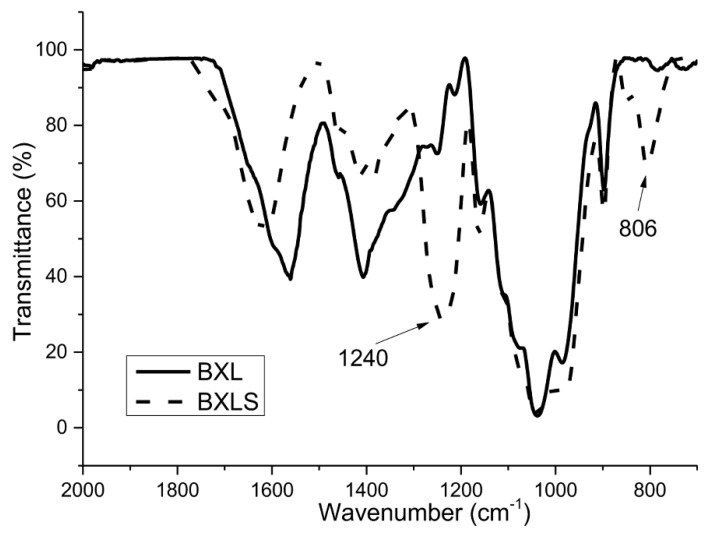
FTIR spectra of non-sulfated (BXL) and sulfated (BXLS) glucuronoxylan samples.

**Figure 2:**
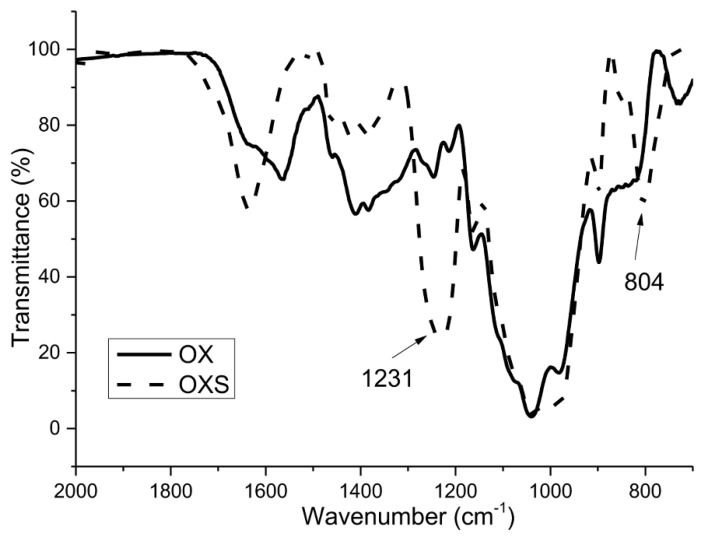
FTIR spectra of non-sulfated (OX) and sulfated (OXS) arabinoxylan samples.

It can be seen clearly from the FTIR spectra in Figure 1 and Figure 2 that two bands typical for the presence of sulfate groups appeared for both sulfated samples.

The spectra for non-modified xylans (BXL and OX) show characteristic peaks for these polysaccharides: The peak at 897 cm^−1^ is typical for the β configuration of C1 in xylans (β-(1→4)), the peak at 1039 cm^−1^, which is assigned to C–OH bending and the peak typical for glycoside linkage appeared at 1160 cm^−1^ [39,40,41,42].

As already mentioned, the success of the sulfation process was confirmed with two bands, which appeared in the spectra of derivatized xylans (BXLS and OXS). The first one, at 805 cm^−1^, is characteristic for the symmetrical vibration of C–O–S, and the second one, in the region of 1200–1260 cm^−1^, is characteristic for the asymmetric valence vibrations of the –SO_2_ or R–SO_3_– groups [43].

According to the results of elemental analyses, which were reported and represented previously [34], both sulfated xylan samples (BXLS and OXS) contained more than 5% of sulfur, which additionally proved the successful introduction of sulfate groups. 

The amount of weak acids determined by potentiometric titration accounted for the sample BXL 0.83 mmol/g, whilst for the sample OX, 0.44 mmol/g [34]. The ratio of carboxylic acidic group content between both reference samples was roughly similar to that determined by 1H-NMR (acidic groups BX: acidic groups OX = 1:2 ≈ 3). The determined pK value of 3.5 agreed with the pK value of glucoronic acids of cellulose [44]. The decrease of pH below this value caused extensive protonation of xylan’s carboxyl groups, and at pH = 2 they became fully protonated (–COOH form). The sulfate groups, as strong acids, are deprotonated in the whole pH region of titration (from pH 2 to pH 11). Based on this knowledge, it was possible to distinguish between carboxyl and sulfate groups in sulfated xylans using polyelectrolyte titration as a function of pH (Table 1).

Titration results at pH 8 showed that the BXL sample contained 0.61 mmol/g acidic groups, which is about 177% higher in comparison to the OX sample acidic groups (0.22 mmol/g) [34]. At pH = 2 the existing total anionic charge belonged to the sulfated groups only and amounted to 1.65 mmol/g for BXLS and 1.39 mmol/g for OXS. With increasing of pH the origin of anionic charge is, besides in the deprotonated sulfated groups, also in the deprotonated carboxyl groups, and the difference in total charge at pH = 8 with the amount of sulfated groups charge at pH = 2 may be related to the amount of dissociable carboxyl groups. 

The anticoagulant activities of sulfated xylans were analyzed by the anticoagulant assay for APTT determination. It can be seen clearly that sulfated xylans prolonged APTT significantly in comparison to non-sulfated samples, and the APTT rose with the increased sulfated xylans concentration (Table 2). 

Non-sulfated BXL and OX samples had no influence on APTT, and there was also no difference in APTT between both samples for the whole concentration range, which indicated clearly that solely the amount of carboxyl groups had no effect on the anticoagulant properties of the samples. Sulfation, however, caused significant enhancement in the anticoagulant activities of xylan samples. Already at small concentrations (5 mg/L) of sulfated xylan samples, APTT increased by about 70% in comparison to the reference samples. No significant differences between sulfated glucuronoxylan and arabinoxylan could be observed at these concentrations. However, at higher concentrations, the differences between both become more explicit, and at the concentration of 15 mg/mL the APTT of the OXS was lower by about 20% than that of BXLS. Obviously, for anticoagulant properties, the sum of both negative charges was decisive, caused by the presence of deprotonated sulfate groups, as well as carboxyl groups (Table 1).

### 3.2. Adsorption Studies

In the adsorption studies, the sulfated xylans were adsorbed onto PET model films at different pH values (pH 4, pH 5, pH 7 and pH 9) and different ionic strengths of monovalent NaCl and divalent salt CaCl_2_ of 0.05 M, 0.1 M, 0.3 M, 0.5 M and 0.7 M. The concentration of xylan’s solutions applied for adsorption procedures amounted to 100 mg/L as optimal, defined in a preliminary research, where the concentrations of 50, 100, 200 and 500 mg/L were tested. 

#### 3.2.1. Influence of pH

Preliminary adsorption studies without electrolyte addition showed no adsorption at all, owing to the same (negative) charge of both the adsorbent (PET surface) and adsorbates (xylans). Thus, 0.1 M CaCl_2_ was applied, in order to screen repulsive forces between the anionic charged PET film and negatively charged carboxyl and sulfated groups of xylans.

According to our earlier investigations [45,46], the frequency changes at the equilibrium of adsorption of non-derivatized xylan samples were −97 Hz for glucuronoxylan and −141 Hz for arabinoxylan. After rinsing with water, the frequency change in the case of glucuronoxylan was around −60 Hz and, for arabinoxylan, around −110 Hz, which resulted in the highest equilibrium adsorbed mass of 2219 ng cm^−2^ for OX at pH 7 (Figure 6). For BXL the highest equilibrium adsorbed mass approached 880 ng cm^−2^ at pH 5. The higher adsorption and stability of OX on the PET surface was most probably driven by its lower solubility in water, coiled conformation, and by the lower repulsion forces between the less negatively charged OX molecules and the PET surface.

**Figure 3:**
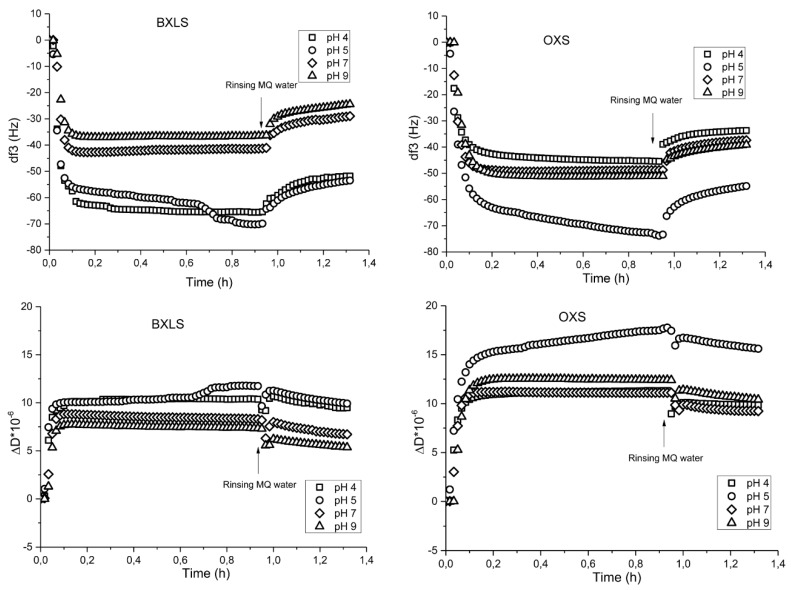
Frequency (third overtone) (**above**) and dissipation (**below**) changes as functions of time during the adsorption of sulfated glucuronoxylan (BXLS) and arabinoxylan (OXS) onto poly(ethylene terephthalate) (PET) model films at different pH values (pH 4, 5, 7 and 9).

Sulfated xylans showed differences in frequencies (df) and dissipation (dD) at different pH values (Figure 3). Prevalent adsorption was observed for both sulfated xylans at pH = 5 with a frequency decrease to about −80 Hz. The dissipation changes were in accordance with the changes in frequencies. The main increase in dissipation as result of a higher deposited amount of sulfated xylans was also observed at pH = 5, associated with incorporated water in the adsorbed xylans layers (Figure 3). 

Rinsing with MQ water did not change the frequencies’ and dissipation’s changes considerably. After the rinsing step for both sulfated samples, the frequency change determined at pH 5 dropped to ca. −56 Hz. Also, at all other pH values, similar trends of desorption occurred (the frequency change decreased by about 20−30%). Such desorption was reasonable, due to the fact that the main driving forces of adsorption are physical interactions (hydrogen bonding and Van der Waals forces) with possible entropy gain adsorption as well, therefore, fully irreversible binding is not expected [47].

#### 3.2.2. Influence of Ionic Strength

For the investigation of the ionic strength’s influences on the adsorption kinetics, two different types of electrolytes, monovalent NaCl and, divalent CaCl_2_ with different molarities (0.05, 0.1, 0.3, 0.5 and 0.7 M) were used with the xylan solution concentration of 100 mg/L and pH 5. pH = 5 was defined as an optimal for adsorption.

**Figure 4:**
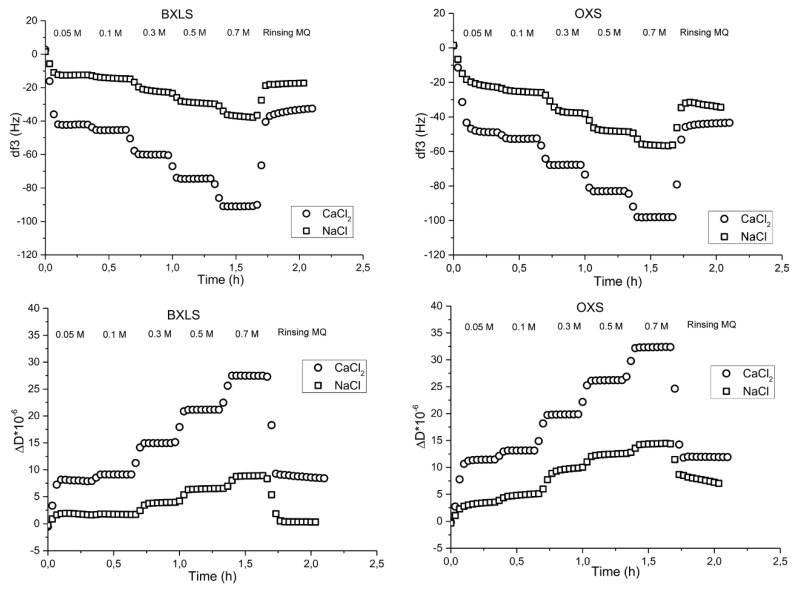
Frequency (third overtone) (**above**) and dissipation (**below**) changes as a function of time during the adsorption of sulfated glucuronoxylan (BXLS) and arabinoxylan (OXS) onto PET model films in the presence of electrolytes (NaCl and CaCl_2_).

With the increase of the ionic strength, the adsorption of sulfated xylans increased, thus, the changes in frequencies increased, and were the largest at 0.7 M (Figure 4). 

These trends were also confirmed for the non-modified reference xylan samples in our previous investigation and are, thus, not shown graphically here [45]. It was demonstrated that OX adsorbed to a higher extent than BXL at each ionic strength, and for both samples the frequencies changes were higher when using a divalent electrolyte (CaCl_2_) in comparison to monovalent NaCl. At the highest CaCl_2_ concentration of 0.7 M, the frequency decreased to around −100 Hz for BXL, and even to −140 Hz for OX, while, at the same concentration of NaCl, the frequency changes were −60 Hz (BXL) and −86 Hz (OX). Owing to the higher −COOH concentration, BXL was more hydrophilic, and its molecules occupied a larger hydrodynamic volume than OX. The effectivity of the added electrolyte was higher in the case of low −COOH content [45]. Oppositely, with a higher amount of deprotonated carboxyl groups, a higher amount of salt is needed to successfully shield negative charges (i.e., repulsion between existing deprotonated carboxyl groups) to ensure a more intense adsorption (due to the collapsed/coiled conformation) on the negatively charged PET surface. The latter is more successful if the electrolyte ion valence is higher whilst besides screening the charge the adding of divalent salt shrinks the electrical double layer more extensively as monovalent salt [46].

Similar trends were observed when sulfated xylans were adsorbed onto PET films (Figure 4); with the increase of ionic strength, the frequencies’ changes were larger because of higher adsorption. Moreover, divalent salt affected the adsorption process more intensively, even at the low concentration level of 0.1 M, while NaCl did not improve the adsorption significantly. At the 0.05 M concentration of monovalent (NaCl) electrolyte, the frequencies changes were −12 Hz for BXLS and −23 Hz for OXS, and at a 0.7 M NaCl concentration, they amounted to −38 Hz for BXLS and −56 Hz for OXS. On the other hand, at the CaCl_2_ concentration of 0.05 M, frequency changes amounted to −42 Hz for BXLS and to −50 Hz for OXS and at the highest CaCl_2_ concentration of 0.7 M, frequency decreased to −90 Hz for BXLS and to −98 Hz for OXS. This was due to the coil-like xylan macromolecular conformation in the presence of CaCl_2_, due to screening the repulsion between existing anionic groups. Moreover, complexes between Ca^2+^ ions and deprotonated sulfated and carboxyl groups of xylan chains were formed [48]. However, after rinsing with MQ water, frequencies increased again, owing to the removal of significant amounts of adsorbed layers.

Comparison of adsorption between reference xylan samples and sulfated xylans clearly suggest that adsorption is to some extent hindered when sulfated xylans are adsorbed onto PET films. In the case of CaCl_2_ addition, the differences in equilibrium frequencies’ changes were lower by about 30% for sulfated xylan samples (BXLS, OXS) in comparison to reference xylans’ samples adsorption (BXL, OX). In the case of monovalent electrolyte NaCl presence, these differences were even higher (about 50%). The reason for such differences may be in the more intensive electrostatic repulsive forces between sulfated xylans and PET film. PET films possess some small amount of anionic charge due to preferential adsorption of OH^−^ from solutions at pH > 3.1 [35], and due to partially deprotonated carboxylic groups at pH = 5, whilst sulfated xylans possess anionic charge due to deprotonated carboxylic groups and sulfated groups (as shown in Table 1). Due to the fact that anionic charge of sulfated xylans is stronger and higher than anionic charge in reference xylans which possess only small amounts of –COOH groups [34], the repulsive forces between the PET surface and sulfated xylan’s molecules are stronger, and thus adsorption poorer. The latter is more pronounced at lower ionic strength and low electrolyte valence, where the screening effect of the repulsive forces between the PET films and sulfated xylans is weaker. With the higher ionic strength of the xylan solutions, the repulsive forces between PET and sulfated xylans were better shielded, and both polymers were able to approach the distance of the Van der Waals forces to be achieved, thus influencing/attracting each other. Hydrogen bonds may also occur due to the presence of hydrogen and electronegative atoms between both polymers (PET and sulfated xylans).

However, all involved physical forces and possible hydrophobic interactions are weak and, therefore, the desorption of adsorbed sulfated xylans’ molecules from PET surfaces after rinsing with water was significant, and higher in comparison to reference samples.

#### 3.2.3. Influence of Anchoring Agents

**Figure 5:**
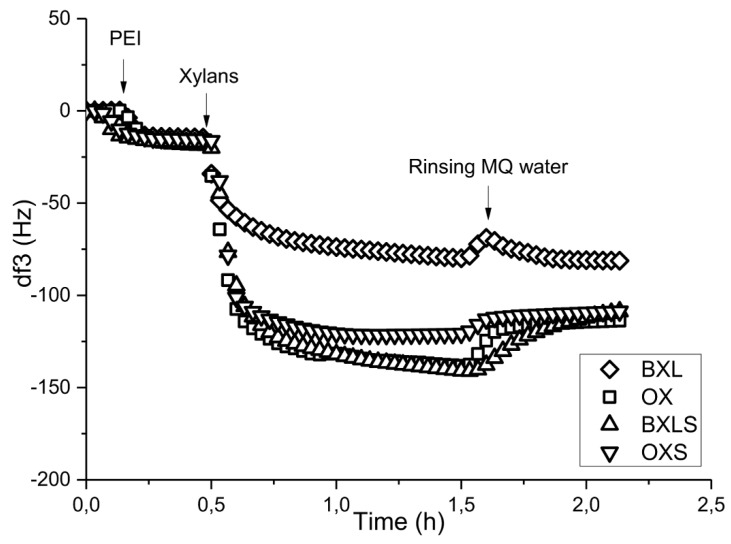
Frequency change at third overtone (df3) as a function of time during adsorption of non-modified and sulfated xylans onto the PET surface after adsorption of polyethyleneimine (PEI) as an anchoring agent.

The positively charged PEI was applied as an anchoring polymer for the adsorption of sulfated xylans onto PET films, with the prediction that positively charged groups of PEI will be created on the PET films surfaces, providing binding sites for sulfated groups of xylan. In this way, strong intermolecular forces such as Coulomb interactions can be achieved, thus improving the stability of coatings. Figure 5 illustrates the frequencies’ changes as functions of time during the adsorptions of non-modified and sulfated xylans using PEI as anchoring layers, previously adsorbed onto PET films from a 0.05% solution and dried. 

This procedure was chosen on the basis of previous investigations, which proved the maximum and stable adsorption of PEI onto PET surfaces at the polymer concentration of 0.05% [49]. In the case of non-modified BXL, frequency change showed the smallest decrease, of about −80 Hz, and, after rinsing with MQ water, the frequency remained almost the same, indicating the adsorbed xylan layer stability. The frequency decrease during the adsorption of arabinoxylan was around −140 Hz, and after rinsing with MQ water, frequency was increased by about 30 Hz, pointing out some desorption of the adsorbed OX. The reason for the differences in adsorption behavior between glucuronoxylan and arabinoxylan could originate from the alterations in their molecular structures [50]. As has already been shown [34], the average molecular mass of arabinoxylan was about 26% higher than that of glucuronoxylan, and its macromolecules were more branched. As such, arabinoxylan formed larger coils, which covered (preferentially adsorption) the positive PEI surface more quickly. On the other hand, the anionic charge density in the case of arabinoxylan is higher and, thus, assures better electrostatic attraction, i.e., Coulomb interaction with the oppositely charged PEI. 

Additionally, the fastest reaching of adsorption equilibrium could be the consequence of the lower hydrophilicity of OX in comparison to BXL, thus, the low water solubility of polymer is the main driving force of adsorption [51].

After the adsorption of sulfated xylans onto PET-PEI films, frequency decreased to about −140 Hz for BXLS and to about −120 Hz for OXS (Figure 5), which is much higher decrease than in the case of reference xylans. Due to higher amount of available anionic charge in sulfated xylans stronger and more extensive electrostatic interaction between PEI, attached onto PET surface, and sulfated xylans were created. Thus, there was just a negligible frequency increase after rinsing with MQ water for both of these xylans because of the minimal desorption.

**Figure 6:**
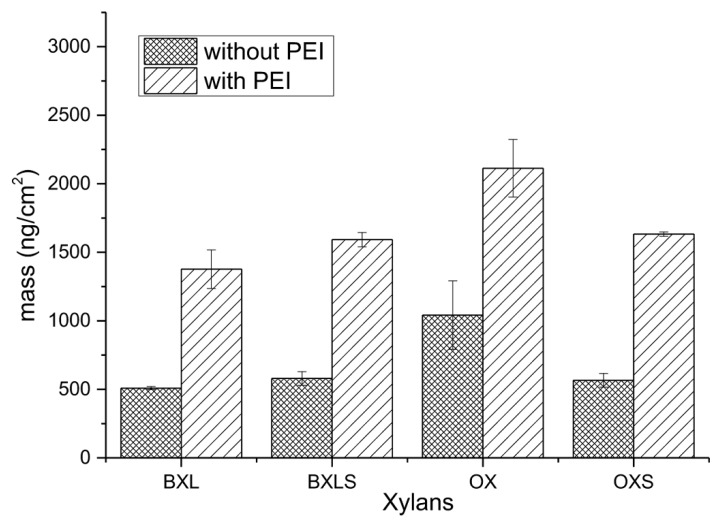
Calculated adsorbed mass of non-modified and sulfated xylans after rinsing with and without preliminary adsorbed PEI as an anchoring agent.

Based on adsorption analyses, the adsorbed equilibrium masses of xylans after rinsing steps with and without the application of an anchoring agent were calculated according to the Equation (1) and are presented in Figure 6. 

The adsorbed masses of anchoring agent (PEI) were subtracted in all cases. Only the masses of adsorbed xylans (together with entrapped water and electrolyte molecules) are taken into account.

From these results, it can be seen clearly that the anchoring agent PEI contributed significantly to a better adsorption of xylans onto PET surfaces. When the anchoring agent was not applied, the masses of adsorbed xylans were significantly smaller in comparison to the masses of adsorbed layers onto the anchoring agent’s layer. 

Equilibrium adsorbed masses of non-modified xylans increased after PEI application by about 103% in the case of OX and 170% in the case of BXL, while the equilibrium adsorbed masses of sulfated xylans increased by 175% for BXLS, and even by 189% for OXS. Besides better adsorption affinity, the electrostatic interactions between PEI amine groups and anionic groups of xylans (carboxylic groups in reference xylans, and both carboxylic as well as sulfate groups in sulfated xylans), also caused significantly better-adsorbed layer stability, proved by the increase in equilibrium adsorbed masses. The cationic charged PEI layer provided enough binding places for anionic groups of adsorbates, enabling stronger electrostatic interactions and, thus, more irreversible attachment of xylans onto PET-PEI surfaces.

### 3.3. X-Ray Photoelectron Spectroscopy XPS

QCM reference and functionalized crystals were, after equilibrium adsorption, dried in a vacuum and further subjected to XPS analysis. In Table 3, the elemental compositions (at. %) of functionalized PET films are given.

It can be seen from Table 3 that, after PEI adsorption onto the PET model film, nitrogen appeared on the surface in the amount of 6.6 at. %, and, at the same time, the surface content of oxygen decreased by about 24% in comparison to its surface content in the PET model film. After non-modified (BXL and OX) xylans’ adsorption, the surface share of nitrogen decreased by about 24%, and, at the same time, the surface content of oxygen increased by about 20%. Such changes in surface chemical composition verified that xylans were adsorbed in the layers thinner than 10 nm (XPS detection zone), as nitrogen from the PEI layer could still be detected.

After the adsorption of sulfated xylans onto PET-PEI surfaces sulfur was introduced, and its surface content was 2.5 at. % or 3.1 at. % after BXLS and OXS adsorption, respectively. In these cases, on the surfaces (10 nm deep), nitrogen could also be detected as the consequence of the PEI presence. In the case of OXS, sulfur content is higher by around 24% in comparison to BXLS. The latter is in accordance with the results of the elemental analysis, which showed the sulfur content in OXS more than 15% higher in comparison to BXLS. It must be pointed out that the elemental composition of the prepared modified surfaces is very promising, while sulfur as a thin outer layer can support the antitrombogenetic effectiveness and nitrogen still available on the surface can contribute to antimicrobial activity. It has been shown in our previous work that amino groups (as the origin of nitrogen) are mainly responsible for pathogen microorganism inhibiting [52].

### 3.4. Wettability and Contact Angle 

After equilibrium adsorption, QCM reference and functionalized crystals were dried in a vacuum, and the wettability of PET surfaces with and without adsorbed PEI and xylans was analyzed using static water contact angles measurements with a goniometer.

**Figure 7:**
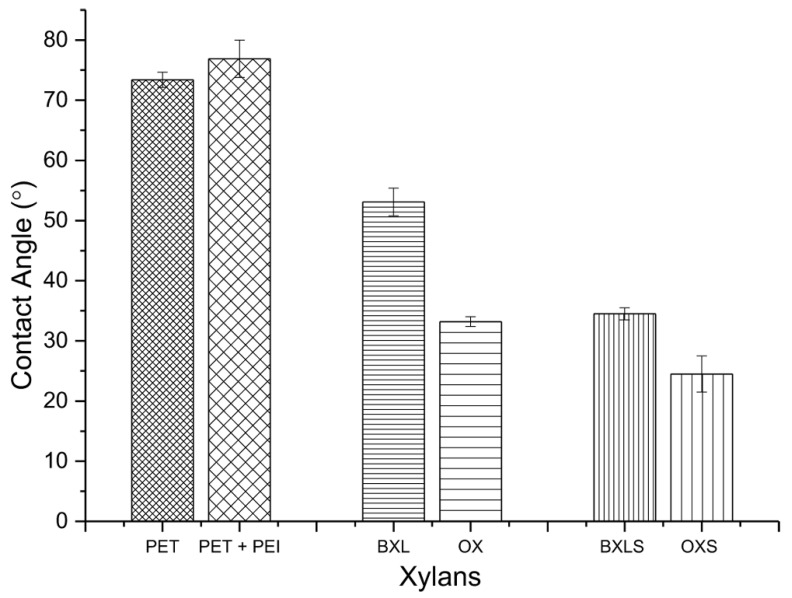
Water contact angles of non-modified PET surface (PET), PET surface covered by PEI (PET-PEI) and PET-PEI surfaces after the adsorption of non-modified xylans (BXL and OX), and sulfated xylans (BXLS and OXS).

The static water contact angles (SCA) of PET surfaces before and after adsorption of PEI and xylans are represented in Figure 7. 

The SCA of PET model film was 73.4 ± 1.3°, and after the adsorption of PEI, the SCA rose to 76.9 ± 3.1°. It is well known that the presence of amino groups influences the surface’s hydrophobicity increase. The introduced carboxyl groups, by the adsorption of non-modified xylans (BXL and OX) onto PET-PEI surfaces, resulted in more hydrophilic surfaces. Water contact angles in the case of PET-PEI covered with non-modified xylans were 53.1 ± 2° for BXL and 33.2 ± 1° for OX, which proved an increase in surface hydrophilicities by about 31% and 55% respectively.

The adsorption of sulfated xylans onto PET–PEI surfaces caused an even larger decrease in SCAs in comparison to adsorbed non-modified xylans. SCAs of sulfated xylans’ surfaces amounted to 34.5 ± 1° for BXLS and 24.5 ± 3° for OXS. Most probably, the reason for such an increase of hydrophilicities by about 55% and 68% for BXLS and OXS respectively, is in the higher amounts of polar carboxylic and sulfate functional groups introduced onto the PET-PEI surfaces via the sulfated xylans. 

These results also proved the good covering of PET-PEI surfaces; thus, they became significantly more hydrophilic.

It is well known that; besides its chemical structure, the surface hydrophobic/hydrophilic character is an important parameter when discussing blood-contacting surfaces. It is a generally accepted theory that a hydrophilic polymer-blood interface is beneficial for reducing platelet adhesion and thrombus formation [53], thus, it is of great importance, through PET chemical surface modification beside antithrombotic function, also to introduce the hydrophilic character. The in vivo evaluation of ammonia plasma-modified ePTFE grafts for the replacement of small diameter blood vessels showed a positive effect of the plasma also due to increased hydrophilicity [54]. 

## 4. Conclusions

The adsorption of sulfated glucuronoxylan and arabinoxylan onto PET model films was studied as a function of the pH and ionic strength of solutions using QCM. To achieve a better persistence of the adsorbed xylan layers, the effectiveness of a polyethyleneimine (PEI) as intermediate anchoring layer was investigated. The hydrophilic/hydrophobic character of the functionalized PET films’ surfaces were examined, as well as their elemental composition. Those properties are extremely important at interfaces of biomaterials and biological environments. 

The basic adsorption studies of non-modified and sulfated xylans onto PET model surfaces using QCM-D showed the increased adsorption at pH 5, as well as under increased ionic strength using divalent calcium cations. The non-modified xylans adsorbed better in comparison to the chemically modified (sulfated) ones, due to lower anionic charge and less extensive repulsive forces between the PET surface and adsorbate macromolecules. When rinsing with water, both types of xylans, reference and sulfated xylans, were desorbed from PET surfaces in rather high amounts. 

The adsorption of both sulfated xylans was increased when, previously, an intermediate layer of chosen functional polymer (so-called anchoring agent—PEI) was applied on the surface of the PET model films. Cationic PEI contributed significantly to better adsorption of sulfated xylans, as well as to their better persistence. This was confirmed by the determination of equilibrium-adsorbed masses after rinsing with MQ water. When the PEI layer was applied, the adsorbed mass of OX increased by about 103% and of BXL by about 170%. In the case of sulfated xylans, the equilibrium adsorbed masses increased by more than 180% in comparison to the adsorption when PEI was not applied. The most important function of the anchoring polymer was the persistence of the adsorbed xylans layers, thus the desorption during the water rinsing was negligible.

The presence of the adsorbed xylans layers on the PET model films surfaces was confirmed by the surface chemical composition analyses and static water contact angles measurements. Adsorption of sulfated xylans introduced significant amounts of sulfur onto the PET-PEI surfaces, which clearly proved their attachment on the PET-PEI model films.

The increase of the surface hydrophilicity was proved by the 55% or 68% decrease of static water contact angles (SCA) for BXLS and OXS adsorbed layers respectively, in comparison with the pure PET-PEI surface.

This research proved that the adsorption of xylans onto PET supported by anchoring polymer PEI resulted in good coating stability, along with hydrophilic and antithrombotic properties caused by sulfate groups content and total anionic charge. 

The defining of adsorption processes through QCM-D model films’ adsorption analyses are a valuable tool, enabling the definition of optimal adsorption conditions, and, in this way, it is extremely supportive in the designing of real systems and new materials’ and products’ development. The QCM-D results of sulfated xylans adsorption onto PET films are essential when planning a functionalization process for real PET blood-contacting medical devices, such as vascular grafts, vascular stents, heart valve cuffs, etc.

## Figures and Tables

**Table 1:** Amounts of deprotonated/protonated acidic groups in sulfated xylan samples (BXLS and OXS) at different pH values.

Xylan Sample	Amounts of Deprotonated Functional Groups [mmol/g]		
	pH8	pH4	pH2
BXLS	3.11 ± 0.52	2.16 ± 0.00	1.65 ± 0.0
OXS	2.43 ± 0.07	2.11 ± 0.00	1.39 ± 0.0

**Table 2:** Activated partial thromboplastine time for different concentrations of non-modified (BXL, OX) and sulfated xylan samples (BXLS, OXS).

Xylan Sample conc. (mg/L)	APTT (s)			
	BX	OX	BXLS	OXS
0	34.8	35.2	34.8	35.2
5	34.8	35.2	51.4	52.1
10	34.8	35.2	64.3	57.8
15	34.8	35.2	98.2	82
20	34.8	35.2	115.9	113.3

**Table 3:** Surface elemental compositions (at. %) of PET model films functionalized with PEI and non-modified and sulfated xylans.

Xylan Sample	C	N	O	S	Si
PET	74.1		25.9		
PET-PEI	71.7	6.6	19.7	0.6	0.9
PET-PEI-BXL	67.1	5.4	26.5		
PET-PEI-OX	63.4	4.6	31.4		0.7
PET-PEI-BXLS	58.1	8.3	30.4	2.5	
PET-PEI-OXS	56.2	8.8	30.6	3.1	

## Abbreviations

PET	Poly(ethylene terephthalate)
QCM-D	Quartz Crystal Microbalance with Dissipation
PEI	Polyethyleneimine
BXL	4-O-methyl glucuronoxylan
OX	Arabinoxylan
BXLS	Sulfated glucuronoxylan
OXS	Sulfated arabinoxylan
SCA	Surface Contact Angles
